# Dynamics of contextual modulation of perceived shape in human vision

**DOI:** 10.1038/srep43274

**Published:** 2017-02-23

**Authors:** Elena Gheorghiu, Frederick A. A. Kingdom

**Affiliations:** 1 University of Stirling, Department of Psychology, Stirling, FK9 4LA, Scotland, United Kingdom; 2 McGill Vision Research, Department of Ophthalmology, McGill University, Montreal, Qc, Canada

## Abstract

In biological vision, contextual modulation refers to the influence of a surround pattern on either the perception of, or the neural responses to, a target pattern. One studied form of contextual modulation deals with the effect of a surround texture on the perceived shape of a contour, in the context of the phenomenon known as the shape aftereffect. In the shape aftereffect, prolonged viewing, or adaptation to a particular contour’s shape causes a shift in the perceived shape of a subsequently viewed contour. Shape aftereffects are suppressed when the adaptor contour is surrounded by a texture of similarly-shaped contours, a surprising result given that the surround contours are all potential adaptors. Here we determine the motion and temporal properties of this form of contextual modulation. We varied the relative motion directions, speeds and temporal phases between the central adaptor contour and the surround texture and measured for each manipulation the degree to which the shape aftereffect was suppressed. Results indicate that contextual modulation of shape processing is selective to motion direction, temporal frequency and temporal phase. These selectivities are consistent with one aim of vision being to segregate contours that define objects from those that form textured surfaces.

In natural scenes, objects rarely occur in isolation, more often than not surrounded by other objects or set against textured surfaces. The human visual system is tasked with extracting and recognizing the shapes of objects from such feature-reach textured backgrounds^1^. Recent psychophysical and computational studies have shown that textures can modulate the perception of the shapes of contours they surround^2^,^3^,^4^,^5^,^6^,^7^,^8^,^9^,^10^. Consider Fig. 1a which shows an anecdotal example from Galli and Zama^11^ of how texture influences the perception of a simple object such as a triangle. When presented in isolation the sides of the triangle are equally salient but when surrounded by a texture (e.g. gratings) the side parallel to the grating bars is perceived as part of the grating/texture not the triangle. We seem unable to perceive the side of the triangle as separate from the grating even when aware that the line is part of a triangle. The related example in Fig. 1b shows that when contours are flanked by several similar-shaped contours it is difficult to perceive the shape of an individual contour. In Fig. 1c, a rectangular shape is partly embedded in a grating, and the embedded parts appear to belong to the grating, as demonstrated by Kanizsa^12^. These simple demonstrations suggest that contours that are parts of textures are processed separately from contours that exist on their own. Put another way, it would appear that contours that are part of textures are prevented from acting as contours that define the shapes of objects.

How might this come about? One idea is that neurons in the visual cortex that possess what has been termed ‘extra classical receptive-fields’ (ERFs) are responsible. A significant number of these neurons are suppressed by textural information that lie outside of their classical receptive field. Specifically, they exhibit what is termed ‘iso-orientation surround suppression’, or IOSS, meaning that the maximum surround suppression occurs when the surround elements are oriented similarly to that of the preferred orientation of the neuron’s classical receptive field^5^,^6^,^8^. These neurons would be expected to be minimally active in response to the contours in Fig. 1 that are surrounded by parallel contours forming a texture, and maximally responsive to isolated contours that define the edges of objects. Recent psychophysical evidence is consistent with the idea that these neurons are implicated in shape perception – for a recent review see Gheorghiu *et al*.^8^. In the context of shape perception the suppressive effect of a texture surround on the perceived shape of a center stimulus has been termed ‘*texture-surround suppression of contour-shape*’, or TSSCS^5^,^6^,^7^,^8^,^13^,^14^. In this communication, we use psychophysics to explore the dynamic properties of TSSCS to further our understanding of contextual effects on shape perception in human vision.

Studies of center-surround interactions in human vision have traditionally employed two protocols: *simultaneous contrast* and *aftereffects*. With simultaneous contrast, the effect of the surround stimulus on the appearance of a center stimulus is measured, as for tilt^15^, size^16^ and density^17^,^18^. With aftereffects, the protocol is somewhat different. An aftereffect describes the change in the appearance of a stimulus following adaptation to a slightly different version of the stimulus. It is a powerful tool for probing visual processes, and has rightly been dubbed the “psychologist’s microelectrode”^19^. When using an aftereffect to study center surround interactions, it is the effect of the surround on the magnitude of the aftereffect induced on the center stimulus that is measured. Thus for example, center-surround interactions in motion processing have been studied using both simultaneous motion contrast^20^ as well as the well-known motion aftereffect^21^ – for a review see Tadin and Lappin^22^. Our interest is in center-surround interactions in shape processing. There are, to our knowledge, no studies of simultaneous shape contrast (we do not include simultaneous tilt or size contrast in this category), whereas a few studies have established that surround shapes can influence the magnitude of aftereffects induced into center shapes^5^,^6^,^23^. For this reason, we have focused on shape aftereffects as a means of studying TSSCS.

The shape frequency aftereffect, or SFAE, is our shape aftereffect of choice. Adaptation to a sinusoidal-shaped contour with a particular shape-frequency (the number of cycles of shape modulation per unit visual angle) causes a shift in the perceived shape frequency of a subsequently viewed test contour with a slightly different shape frequency^5^,^6^,^24^,^25^,^26^. Readers may experience the aftereffect in Fig. 2. If one moves ones’ eyes back and forth along the marker between the pair of adapting contours in Fig. 2a for about a minute, and then shift one’s gaze to the spot in Fig. 2d, the two test contours above and below the fixation dot appear to have a different shape frequency, even though they are physically identical. The shape-frequency aftereffect is an effective tool for probing contour shape processing in human vision, on the assumption that it results from changes in the response distribution of neurons specialized for the coding of contour shape, in a manner similar to that proposed for other spatial aftereffects^27^,^28^.

How then can the SFAE be used to study TSSCS? When the adaptor contour is surrounded by a texture made of similar contours, as in the example shown in Fig. 2b, the magnitude of the SFAE is reduced^6^. The reduction in shape aftereffect is in one sense remarkable, given that the surround contours are all potential shape adaptors. In keeping with the interpretation of the demonstrations in Fig. 1, the surround texture inhibits the processing of the adaptor contour as a *contour*, instead favoring the processing of the adaptor contour as part of a *texture*. Coupled with the idea that contours and textures are processed by different mechanisms, it follows that an adaptor contour that is encoded as part of a texture will have little adaptive effect on a contour that is processed as a contour.

Although several recent studies have examined the various spatial and photometric (luminance and color) properties of TSSCS and the role they serve^7^,^8^,^13^,^14^, little or nothing is known about their temporal- and motion-tuning properties. Timing likely affects the strength of suppression, so an understanding of the dynamics of the process is important for understanding the functional role TSSCS plays in vision. In this communication, we provide new evidence concerning the global and local motion-direction tuning, temporal tuning and temporal phase properties of contextual modulation of shape.

In different experiments, we investigate the dynamics of TSSCS by examining its dependency on motion direction and timing. To do this, we have measured the SFAE with a center contour adaptor and a surround texture that were either the same or different in one of a number of temporal attributes, specifically motion direction, temporal frequency and temporal phase. The premise is that if the SFAE is significantly greater for different-attribute compared to same-attribute center-surround adaptor combinations, then TSSCS mechanisms are tuned to that temporal attribute.

Our stimuli consist of contours and textures made of strings of Gabor elements - these are small patches of sinusoidal gratings windowed by a smooth Gaussian envelope (see Supplementary Information: Appendix A). In the motion direction tuning experiment, the string of Gabors moved in unison as a rigid shape, a type of motion termed *global motion*. The center contour and surround texture in the adaptor were separated by a small gap (0.6 deg) to prevent them from spatially overlapping when they drifted in different motion directions and/or with different speeds (see Fig. 2b,c). In all other experiments, there was no gap. There were three adaptor conditions: center contour only, i.e. no surround (Fig. 2a), center and surround moving in the same direction (Fig. 2b), and center and surround moving in opposite directions (Fig. 2c). The test stimuli in all conditions were pairs of single contours drifting in the same motion direction and with the same temporal frequency as the center contour adaptor (Fig. 2d). Example dynamic stimuli are illustrated in the Supplementary information (Appendix B: Movies S1,S2,S3). We varied the temporal frequency (i.e. 0.9, 1.8, 2.7 and 3.6 shape cycles/sec) of both center and surround contours. This resulted in 16 combinations of center/surround temporal frequencies (4 center × 4 surround temporal frequencies) that were measured for each of the three center/surround motion direction conditions (i.e. 4 no surround, 16 same and 16 opposite center-surround directions conditions), resulting in 36 conditions in total.

To measure the SFAE we used a cancellation method in which subjects adjusted, using a conventional psychophysical procedure, the relative shape frequencies of two test contours that had been adapted using adaptors that differed by a factor of three in shape frequency^24^,^25^,^29^. On each trial, subjects indicated via a button press whether the upper or lower test contour had the higher perceived shape-frequency and the computer then changed the ratio of test shape-frequencies in a direction opposite to that of the subject’s response, until the two test contours appeared equal in shape frequency, i.e. were at the point-of-subjective equality, or PSE (see Methods for details). The magnitude of the aftereffect was then calculated as the physical ratio of the two test shape-frequencies at the PSE.

## Results

### Experiment 1: Motion direction and temporal frequency tuning of surround suppression

In this experiment, we examine the global motion-direction selectivity of TSSCS as a function of shape temporal frequency. If TSSCS is selective for global motion-direction, one predicts that the aftereffects will be released from suppression when the adaptor and test contours move in opposite as opposed to the same directions.

Figure 3a shows the size of the shape aftereffect for three observers plotted as a function of the temporal shape frequency of the adaptor’s texture-surround, for same (gray symbols) and opposite (black symbols) center vs. surround motion directions, and for adaptor/test center contours drifting at 0.9, 1.8, 2.7 and 3.6 shape cycles/sec (as also indicated by the red arrows). The across-observer average shape frequency aftereffect is shown in Fig. 3b. The dashed lines indicate the size of the aftereffect obtained with no adaptor texture-surround. The results show that the aftereffect is strongly reduced when the adaptor surround moves in the same but not opposite direction to the center contour (compare gray and black symbols) suggesting that the surround suppression is selective to global motion direction. One can also see that the reduction in the shape aftereffect increases in magnitude with the temporal frequency of the adaptor’s central contour (compare left to middle to right panels in Fig. 3). This may be related to the fact that in the absence of a texture surround, the size of the aftereffect increases with temporal frequency (compare left to right panels in Fig. 3).

Another way to express the results is in terms of the magnitude of surround suppression. To do this we calculate a surround suppression index (SSI), as follows: *SSI *= 1 − *SFAE*_*WITH−SURROUND*_/*SFAE*_*NO−SURROUND*_, where *SFAE*_*WITH−SURROUND*_ and *SFAE*_*NO−SURROUND*_ are the shape frequency aftereffects (SFAE) obtained with and without the surround texture, respectively. With this metric, 1 indicates complete suppression of the aftereffect while 0 indicates no suppression. Figure 4a shows the across-observers average SSI as a function of texture-surround temporal frequency for different center contour temporal frequencies (see left to right panels in Fig. 4), while Fig. 4b shows the same SSI data plotted in terms of the ratio of center-to-surround temporal frequencies. These results show that for same center-surround motion directions the SSI increases with the temporal frequency of the center contour, from an SSI of 0.33 for 0.9 shape cycles/sec to an SSI of 0.658 for 3.6 shape cycles/sec. With opposite center-surround motion directions, the SSI is lower and invariant to temporal frequency (on average ~0.16).

For each central contour adaptor/test temporal frequency, the data for the shape aftereffect (Fig. 3a) were submitted to a 2 (motion directions: same vs. opposite) by 4 (surround temporal frequencies) repeated measures ANOVA on both factors. The ANOVA showed a statistically significant main effect of global motion direction for all central contour-adaptors temporal frequencies (F(1,2) = 161.2, p = 0.0061 for 1.8 shape cycles/sec; F(1,2) = 654.8, p = 0.0015 for 2.7 shape cycles/sec; F(1,2) = 41.11, p = 0.0235 for 3.6 shape cycles/sec) except for the lower temporal frequency of 0.9 shape cycles/sec (F(1,2) = 14.59, p = 0.0622). For each of the central contour adaptor/test temporal frequencies, the main effect of surround temporal frequency was not statistically significant (p > 0.05) except for the 2.7 shape cycles/sec central contour adaptor/test (F(3,6) = 7.03, p = 0.0217).

Finally, we submitted the data to a repeated-measures one-way ANOVA to examine the effect of central contour adaptor/test temporal frequency for both same (compare gray symbols across left-to-right panels in Fig. 3a) and opposite (compare black symbols across left-to-right panels in Fig. 3a) motion directions. The effect of central contour temporal frequency was found to be significant for the opposite motion direction (F(3,9) = 8.905, p = 0.0047) but not for the same motion direction (F(3,9) = 2.223, p = 0.1548).

### Experiment 2: Local motion direction tuning of surround suppression

To compute the global motion direction of an object the visual system must integrate across space *local* motion signals provided by motion-direction-selective neurons in V1^30^,^31^. Surround suppression of V1 neurons has been shown to be selective to motion direction^32^,^33^. Since in Experiment 1 we found that TSSCS is selective to global motion direction, this raises the question as to whether TSSCS is selective for *local* motion-direction. If TSSCS is mediated by ERF neurons in V1 selective to local motion-direction then we predict a release from surround-suppression when the local motions of the center contour adaptor and surround texture go in opposite directions.

To examine the selectivity of TSSCS to local motion-direction we use contours and textures made of strings of Gabor elements in which the Gabor’s luminance grating, or carrier, moves inside its stationary Gaussian envelope, i.e. when the overall shape of the contour does not move. The carriers were set to drift with velocities that depended on their position within the shape in order that their motions simulated a rigid moving body. Specifically, the velocity of each carrier was defined as the product of the luminance temporal frequency of the carrier and the cosine of the Gabor’s orientation with respect to vertical. Thus, the carrier of a Gabor oriented tangentially to the peak or trough of the shape was stationary, whereas one passing through the d.c. of the shape moved the fastest. This resulted in a perceived global motion that was either leftward or rightward. We used carrier temporal frequencies of 1.25, 2, 3.15, 5, 7.94, 12.6 and 20 luminance cycles/sec, all of which produced coherent motion. The conditions were similar to those of Experiment 1. The carrier temporal frequency of the center contour adaptor and the contour test was fixed at 5 luminance cycles/sec (see red arrows in Fig. 4) for all surround and no-surround conditions. The carriers of the test contour Gabors in all conditions drifted in the same motion direction as those of the center contour adaptor. We also ran a no-surround condition in which the carrier of the central contour adaptor and test moved in opposite directions, in order to examine whether contour shape mechanisms are selective to local motion direction.

Figure 5 shows the aftereffect for four observers (Fig. 4a) as well as the average across observers (Fig. 5b), with SSIs shown in Fig. 5c. Aftereffect size is plotted as a function of the carrier temporal frequency of the surround adaptor, for the same (gray symbols) and opposite (black symbols) center vs. surround motion directions. The blue and black dashed lines are for the no surround same, and no surround opposite adaptor vs. test motion directions. Taking the last of these first, the no surround aftereffect is similar for the same and opposite adaptor vs. test motion directions. For the with-surround conditions the reduction in aftereffect is of similar magnitude for both same and opposite center vs. surround local motion directions (compare gray and black symbols: averages are 0.42 and 0.44 respectively), suggesting that texture surround suppression is not selective to local motion direction.

To confirm the lack of selectivity to local motion direction and to surround carrier temporal frequency, the data for the shape aftereffect shown in Fig. 5a were submitted to a 2 (same vs opposite motion directions) by 7 (surround carrier temporal frequency) repeated measures ANOVA. There was no statistically significant effect of local motion direction (F(1,3) = 0.1318, p = 0.7407), no statistically significant effect of surround carrier temporal frequency (F(6,18) = 2.589, p = 0.0548) and, no statistically significant interaction of local motion direction and temporal frequency (F(6,18) = 1.617, p = 0.1996).

### Experiment 3: Temporal phase-relationship between center and surround

Is the influence of the texture surround selective to the temporal phase relationship between center and surround? If it is, then we would expect strong suppression of the aftereffect for in-phase center and surround and weak suppression for anti-phase center and surround. Given that in Experiment 1 we found that the strength of surround suppression increases in magnitude with the temporal frequency of the adaptor’s central contour, we examined the degree of TSSCS temporal phase selectivity as a function of temporal frequency.

In this experiment, the adaptor surround, the adaptor central contour and the test contour all drifted in the same motion direction and at the same temporal frequency (see Fig. 6a). The new feature is that we set the *contrasts* of the Gabor micropatterns to counter-phase over time. The micropatterns in the center and surround adaptor were counter-phased either in-phase or anti-phase, at temporal frequencies 0, 0.16, 0.33, 0.66, 1.32, 2.64, 5.28 and 10.5 Hz. A schematic of the arrangement is shown in Fig. 6b,c. There were three center and surround adaptor conditions: no surround, center and surround contrast modulated in-phase (Fig. 6b) and, center and surround contrast modulated in anti-phase (Fig. 6c).

Figure 7 shows the results for three observers (Fig. 7a), and their averages (Fig. 7b), as a function of the temporal frequency of the contrast modulations, for the in-phase (white symbols), anti-phase (gray symbols) and no-surround (black symbols) conditions. Average across-observer SSIs are shown in Fig. 7c. From this figure one can see that, overall, surround suppression for the in-phase condition was strong and about the same (~0.82) across temporal frequency, whereas for the anti-phase condition surround suppression was very small at the lowest temporal frequency, increasing monotonically with temporal frequency until reaching more-or-less the same level as the in-phase condition at the highest temporal frequency.

The data (Fig. 7a) were submitted to a 2 (temporal phase-relationship) by 7 (temporal frequency) repeated measures two-way ANOVA. The analysis showed a statistically significant effect of temporal phase-relationship (F(1,12) = 44.96, p = 0.0215) and of temporal frequency (F(6,12) = 3.01, p = 0.0493). Finally, there was a statistically significant interaction between phase-relationship and temporal frequency (F(6,12) = 4.985, p = 0.0088) indicating that the effect of phase-relationship changed as a function of temporal frequency. To investigate this interaction, we examined whether the slope of the linear regression line that relates aftereffect magnitude to temporal frequency for the in-phase and anti-phase conditions were different. The analysis revealed that the differences between the slopes were statistically significant (F(1,10) = 72.42, p < 0.0001).

### Experiment 4: Temporal phase-difference between center and surround

In the previous experiment we found that the strength of surround suppression increases monotonically with temporal frequency for the anti-phase condition. Here we examine more closely the temporal-phase selectivity of TSSCS by varying the temporal-phase difference between central contour and surround texture adaptor. We expect strong suppression of the aftereffect for small temporal-phase differences between center and surround and a release from suppression for larger temporal-phase differences between center and surround.

All contrast modulations were at 1.32 Hz (an intermediate temporal frequency for which surround suppression was of intermediate strength in Experiment 3). The adaptor was not coherently moving but its shape-phase was randomly changed every cycle of contrast modulation (i.e. 1.32 Hz) in order to prevent the formation of afterimages and to minimize the effects of local orientation adaptation. The shape-phase of the test contour was also randomly assigned in every test period. We varied the temporal-phase difference between the central contour and surround texture adaptor. We used eleven values of temporal phase difference: 0, ±15, ±30, ±45, ±60 and ±90, with the ‘+’ sign indicating that the central contour preceded the surround texture and the ‘−’ sign indicating that surround texture preceded the central contour. A schematic of the phase difference condition is shown in Fig. 6d.

Figure 8a shows the aftereffect as a function of the center-surround temporal phase difference for three observers and their average (Fig. 8a). The average SSIs are shown in Fig. 8b (the data in the right panel is the same as shown in the left panel but disregarding the sign of the phase difference). The black dashed lines indicate the no-surround condition and the blue vertical dashed line the condition in which center and surround were in temporal phase. As expected, the surround suppression peaked around zero center-surround phase difference. The decline in surround suppression either side of zero is gradual, showing that the suppression is broadly tuned for center-surround temporal phase differences. There is a hint of an asymmetry in the suppression, with suppression being somewhat larger when the surround texture preceded rather than followed the central contour (see right panel in Fig. 8b) the maximum suppression was obtained for a temporal phase difference of −15 which corresponds to ~50 ms time difference between center and surround.

The data (Fig. 8a) were submitted to a 5 (phase difference) by 2 (center-surround temporal order or the sign +/− of phase difference) repeated measures ANOVA on both factors. The ANOVA showed a statistically significant main effect of phase difference, F(4,8) = 14.62, p = 0.0009, indicating that the aftereffect decreased with phase difference. The main effect of center-surround temporal order was not statistically significant, F(1,2) = 5.204, p = 0.1501, indicating that the aftereffect was not significantly affected by the center-surround temporal order when averaged over phase differences. Finally, there was no statistically significant interaction of phase difference and center-surround temporal order, F(4,8) = 1.157, p = 0.3973, indicating that the effect of phase difference did not changed as a function of center-surround temporal order.

## Discussion

To our knowledge this is the first study to investigate the motion-direction tuning and temporal properties of center-surround suppressive interactions in shape processing. Our results show that for globally drifting contours, the suppression of the shape aftereffect was maximal when the surround and center adaptor contours were moving in the same direction, at the same speed, and at relatively high speeds. On the other hand, for locally moving stimuli, the suppression was unaffected by the relative motion direction of center and surround. When the contrasts of the contour micropatterns were set to counter-phase, the amount of surround suppression was agnostic to counter-phase temporal frequency when center and surround were in-phase, but increased with counter-phase temporal-frequency when in anti-phase. Finally, again using counter-phasing micropatterns, the surround suppression was broadly tuned for center-surround phase differences, and slightly larger when the surround texture preceded rather than followed the central contour. Altogether, these findings have revealed that center-surround interactions in contour shape processing are tuned to global not local motion-direction and temporal frequency, and tuned to the temporal phase-relationships between center and surround.

### Significance for the functional role of surround suppression

These results are in keeping with an important functional role of surround suppression, namely to facilitate the segregation of contours that are part of objects from those that are part of textures. A contour that is part of a texture is unlikely to exhibit different dynamic properties from its neighboring contours, and as a result surround suppression will tend to be maximal, preventing the contour from being processed as part of an object. On the other hand, a contour that is embedded in a texture but which exhibits different dynamic properties from its surround, i.e. is likely to be part of an object rather than the texture, will be minimally suppressed, with the result that its shape will be processed as part of the object to which it belongs. It should be emphasized that this functional account of surround suppression relates only to the processing of object contours. The shapes of textures are processed in a separate stream^12^.

### Relation to previous psychophysical studies on aftereffect dynamics

Motion direction selectivity has previously been found for the shape-frequency aftereffect on its own, i.e. with no surround^34^ as well as for other spatial aftereffects such as the tilt aftereffect^35^,^36^ and the size, or spatial-frequency aftereffect^37^. Motion direction selectivity has also been found for the tilt illusion^36^,^38^. As a footnote to the tilt illusion finding, we have previously provided evidence that the shape-frequency aftereffect is not a manifestation of the tilt aftereffect, i.e. is not caused by orientation adaptation^25^,^34^. Rather, our evidence points to local curvature being the adaptable feature in the shape-frequency aftereffect^14^.

### Neural mechanisms for motion-direction and temporal-phase tuning of TSSCS

Neurophysiological studies have shown that extra-classical receptive field, or ERF neurons, i.e. those subject to influences from outside of their classical receptive fields, or CRFs, are found in many visual areas, such as V1 and V2^39^,^40^,^41^,^42^,^43^,^44^,^45^,^46^,^47^, V4^48^,^49^, MT^50^,^51^,^52^ and MST^53^. For V1 neurons sensitive to oriented lines or luminance gratings exhibiting ERFs, the suppression is maximal when the surround orientations are the same as the preferred orientation of the CRF^32^,^39^,^40^,^41^,^42^,^43^,^54^,^55^,^56^, a phenomenon known as ‘iso-orientation surround suppression’ or IOSS. We have suggested^5^,^6^,^7^,^8^,^14^ that these ERF V1 neurons might mediate the suppression of contour-shape aftereffects, by feed-forwarding their responses to intermediate-to-higher level visual areas known to be involved in contour shape processing, such as V4^5^,^14^,^57^. Neurophysiological studies have also shown that for a subpopulation of V1 neurons (43% cells), the suppression is maximal when the surround gratings drift at 3 Hz in the same direction as the preferred motion direction of the CRF and is minimal when surround motion direction is opposite to that of the CRF^32^. These cells are said to manifest ‘iso-motion-direction surround suppression’. In another subpopulation of V1 neurons (22% cells) there is no reduction in the degree of suppression with motion direction, that is, surround suppression is not selective to motion direction^32^. Therefore, our finding that TSSCS is selective to global but not local motion direction might be the result of different subpopulations of V1 neurons with ERFs that feed-forward their iso-motion-direction surround-suppression responses into shape-selective neurons in areas such as V4. However, it is possible that the suppression takes place directly in the higher visual areas (e.g. V4): some indirect evidence comes from our previous work showing that the suppression only occurs when the surround texture and central adaptor contour are located in the same stereoscopic depth plane^14^.

While some neurophysiological studies report that V4 neurons manifest no selectivity for motion-direction^58^,^59^, others find that non-directional V4 neurons can acquire motion-direction selectivity after adaptation^60^, as a result of feedback from higher areas such as MT and MST. Thus, the global motion-direction and temporal frequency selectivity of TSSCS found in Experiment 1 might be caused by feedback from MT/MST. Our motion-direction results are also consistent with center-surround motion interactions observed in other visual areas such as V1^32^,^61^, MT^50^,^51^,^62^,^63^,^64^,^65^ and lateral MST^53^. Neurophysiological studies have shown that responses of MT neurons are maximally suppressed when the motion direction in the surround is identical to that of their CRF preference^50^,^65^. This squares with our finding that the shape aftereffects were reduced by surround contours moving in the same but not opposite directions to the central contour. Therefore, we suggest the cortical route described above as underpinning our results: V1 neurons with ERFs feed-forwarding their responses into shape-selective neurons in areas such as V4, with feedback from MT and/or MST.

With regard to temporal dynamics, most neurophysiological studies have measured the contrast response functions of V1 neurons using luminance gratings drifting at various temporal frequencies, and find strongest suppression of V1 CRF responses from high temporal frequency surrounds^66^,^67^. Those neurophysiological studies that have examined explicitly the time lag/delay between center and surround responses have used reverse correlation techniques, and find that the suppressive surround effects in V1 neurons peak at around 80 ms after the stimulus onset^68^ with the ERF suppression lagging by up to 60–100 ms^41^,^42^,^44^,^68^,^69^,^70^,^71^. For area MT neurons, the inhibitory component of the surround response was found to lag ~10 ms after the center response^72^. However, it remains to be established whether these time lags reflect time differences in response due to feedback, feedforward or lateral connections within the ERF. Taken together, the temporal dynamics of surround suppression measured neurophysiologically are commensurate with the results of the present study.

### Summary and Conclusion

We found that for globally drifting contours, TSSCS was maximal when the surround and center adaptor contours were moving in the same direction, at the same speed and at relatively high speeds, while for locally moving stimuli, the suppression was unaffected by the relative motion direction between center and surround. In addition, TSSCS was found to be selective for temporal frequency and temporal phase, and broadly tuned to the temporal phase-difference between central contour and surround texture. These motion-direction and temporal tuning characteristics of surround suppression are consistent with the center-surround motion interactions observed in visual areas such as V1^32^,^61^, MT^50^,^51^,^62^,^63^,^64^,^65^ and lateral MST^53^. Taken together our results suggest the involvement of both feed-forward and feedback pathways: V1 neurons with ERFs feed-forwarding their responses into shape-selective neurons in areas such as V4, with feedback from MT and/or MST. These dynamic properties of TSSCS play an important role in extracting contours that define objects from those that define textured surfaces (i.e. it de-texturizes the images^8^). Thus, TSSCS represents an important neural substrate underlying efficient figure (object)-ground (texture) segregation.

## Methods

### Participants

Eight observers participated in this study, the two authors and six observers who were naive with regard to the experimental aims. Three participants took part in Experiment 1, 3, 4 and four in Experiment 2. All observers had normal or corrected-to-normal visual acuity. Observers gave their written informed consent prior to participating in this study and were treated in accordance with the Declaration of Helsinki. The research protocol was approved by the University of Stirling Ethics Committee and McGill University Health Centre Research Ethics Office.

### Stimuli

The stimuli were generated by a ViSaGe video-graphics card (Cambridge Research Systems, UK) with 12-bits contrast resolution, presented on a calibrated, gamma-corrected Sony Trinitron monitor, at 120 Hz frame rate and with a spatial resolution of 1024 × 768. The mean luminance of the monitor was 40 cd/m^2^. Viewing distance was 100 cm.

**Figure 2:**
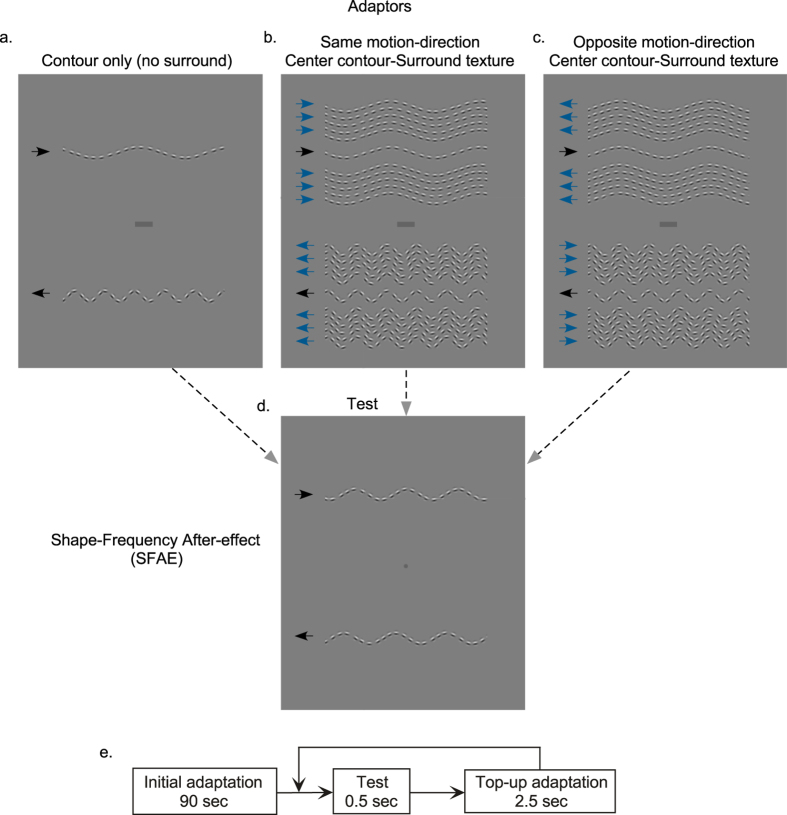
Stimuli used in Experiment 1. One can experience the shape-frequency aftereffect obtained with single contour adaptors (**a**) and textures (**b**,**c**) by moving one’s eyes back and forth along the markers located midway between the pair of adapting contours (top) for about 90 s, and then shifting one’s gaze to the middle of the single test contours (**d**). The textures consisted of a central contour flanked by same orientation surround moving either in the (**b**) same or (**c**) opposite motion direction. A small gap of 0.6 deg was used between center contour and surround texture in order to avoid spatial overlap when center and surround drifted in opposite directions. (**e**) schematic representation of the adapting and test procedure - see text for details.

**Figure 3:**
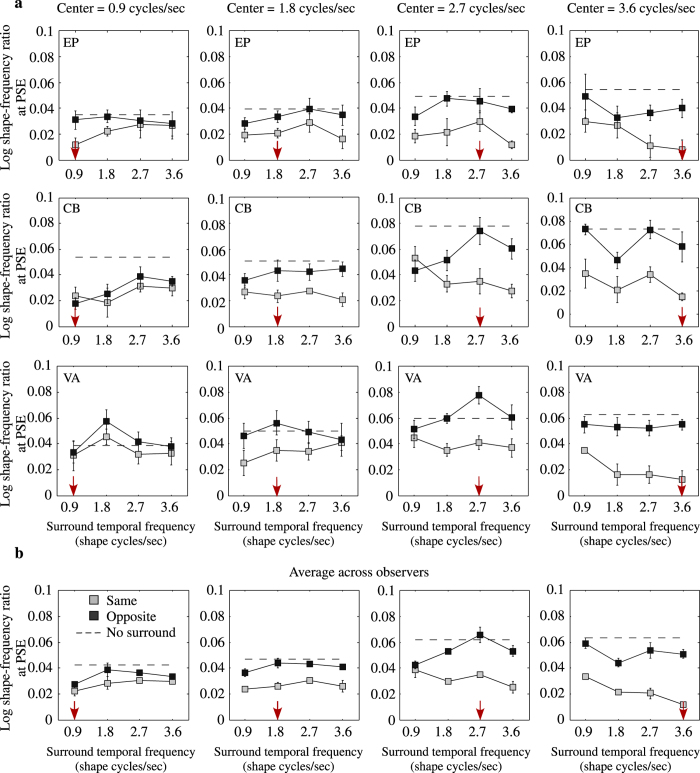
The shape aftereffect plotted as a function of texture-surround temporal frequency for the same (gray symbols) and opposite (black symbols) center-surround motion direction conditions for central contour adaptor-test drifting at 0.9 (left), 1.8, 2.7 (middle) and 3.6 shape cycles/sec (right) for three individual observer (**a**) and the average across-observers (**b**). Dashed lines indicate the size of aftereffects obtained with contour only (or no surround adaptors).

**Figure 4:**
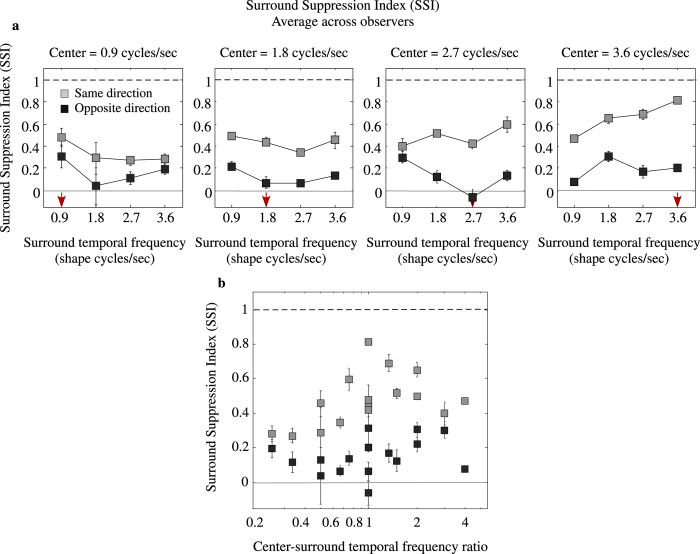
(**a**) Across-observers average surround suppression index (SSI) plotted as a function of temporal frequency of the surround for central contour adaptor-test drifting at 0.9 (left), 1.8, 2.7 (middle) and 3.6 (right) shape cycles/sec. Same SSI data plotted in terms of center-surround temporal-frequency ratio. Note that SSI is inversely related with the magnitude of SFAE. A surround suppression index of 1 indicates complete suppression while 0 indicates complete lack of suppression.

Adapting stimuli were pairs of sinusoidal-shaped textures and contours made of strings of Gabor elements and presented in the center of the monitor on the mean luminance background and located 4 deg above and below the fixation marker. The upper and lower adapting and test stimuli were set to drift in opposite directions, as shown in Fig. 2a–c. The adapting pair consisted of textures or contours with shape frequencies of 0.2 and 0.6 c/deg, giving a geometric mean shape-frequency of 0.35 c/deg. The mean shape-frequency of the test contour pair was held constant at 0.35 c/deg. The shape-amplitude of the two adaptors and tests was fixed at 0.3 deg. All contours were constructed from strings of odd-symmetric (d.c. balanced) Gabor patches with a spatial bandwidth of 1.5 octaves, a center luminance spatial frequency of 4 c/deg and a contrast of 0.85. The Gabor patches were positioned along the sinusoidal-shaped profile and were oriented tangentially to the path of the contour. The center-to-center spacing between adjacent Gabor patches along the contour was randomly selected from within the range ±0.15 deg around a mean of 0.4 deg.

**Figure 5:**
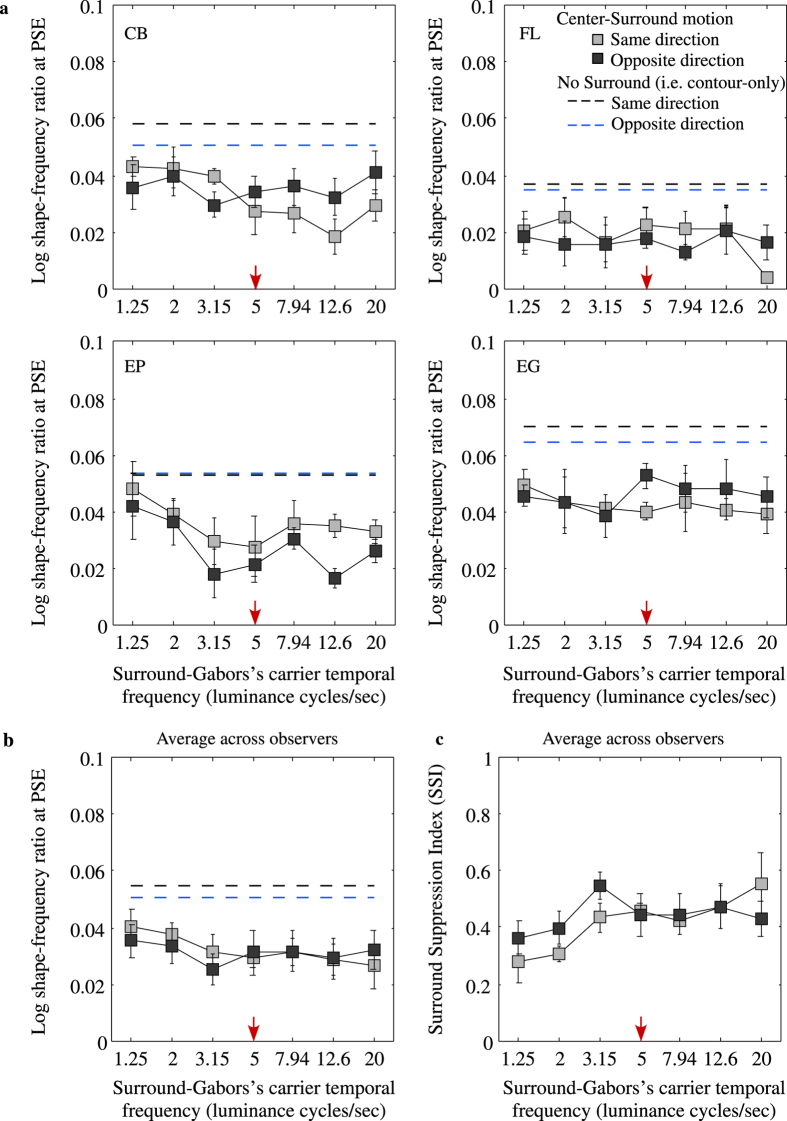
Individual observers (**a**) and average across observers (**b**) shape frequency aftereffect plotted as a function of the luminance temporal frequency of the carriers of the surround adaptor for the same (gray symbols) and opposite (black symbols) center-surround motion direction conditions. The same and opposite local motion-direction conditions for the no-surround (or contour only) adaptor and test conditions are shown by the blue and black dashed lines, respectively. (**c**) The corresponding across-observers average surround suppression index (SSI).

**Figure 6:**
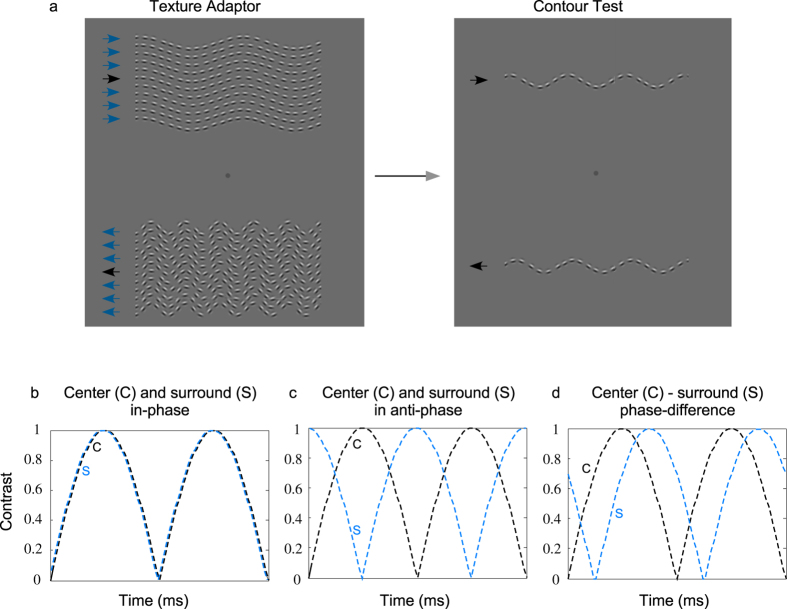
(**a**) Example of a pair of adaptor textures made of a central contour flanked by same orientation surround texture. The test was always a single contour. (**b**,**c**) A schematic of how the contrast of the center-contour and surround-texture adaptor was temporally modulated (**b**) in-phase, (**c**) in anti-phase, and (**d**) with various temporal phase differences.

**Figure 7:**
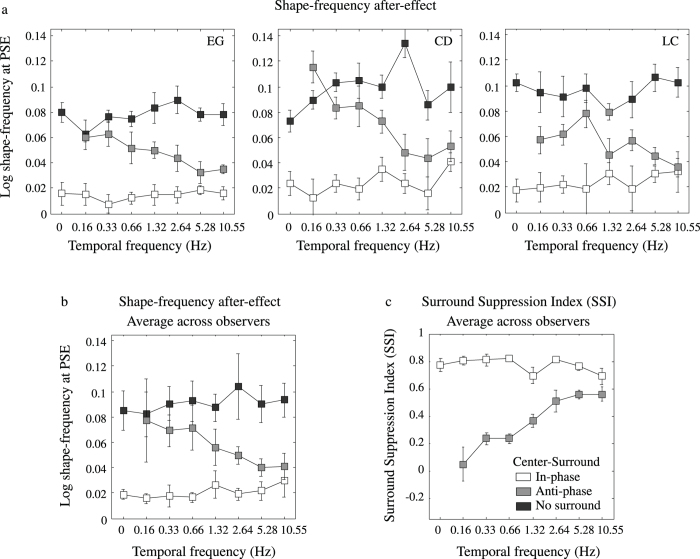
The shape aftereffect for three observers (**a**) and the average across-observers (**b**) plotted as a function of temporal frequency of center-surround contrast modulations for the in-phase (white symbols), anti-phase (gray symbols) and no surround (black symbols) conditions. The average across-observers surround suppression index (SSI) is shown in (**c**).

**Figure 8:**
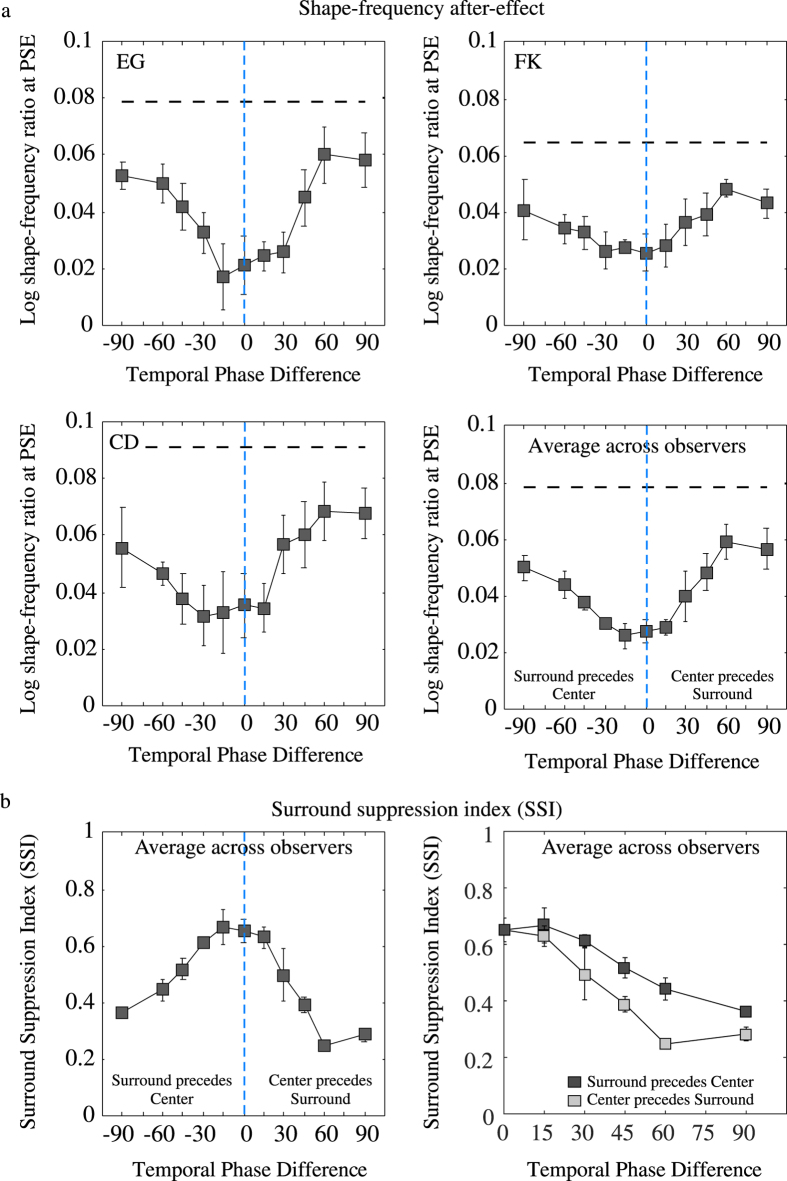
The shape-frequency aftereffect plotted as a function of temporal phase-difference between center and surround adaptor for three observers (**a**) and the average across-observers (**b**). The black dashed lines indicate the shape frequency aftereffects obtained with contour only adaptor (i.e. no surround) and the blue vertical dashed line indicate the condition in which center and surround are in temporal phase. The average across-observers SSI is shown in (**c**).

The texture adaptors consisted of a central contour flanked on each side by a surround made of 5 contours arranged in parallel (i.e. the entire texture contained a total of 11 contours). The center contour and surround texture were separated by a small gap of 0.6 deg which was introduced in order to avoid the spatial overlap between contours when they were made to drift at different temporal frequencies in the same or in different directions (see Fig. 2b,c). This small separation of 0.6 deg between center and surround, which was twice the amplitude of the shape modulation, was used only in Experiment 1. In all other experiments there was no gap between center contour and surround texture (see example in Fig. 5a and/or our previous studies^5^,^6^,^7^,^8^). In Experiment 1, the entire sinusoidal-shaped contours and textures made of Gabor elements moved inside a virtual window. This type of motion in which both the carrier (i.e. luminance gratings) and Gaussian envelope of the Gabor elements drift together is termed *global motion*. In Experiment 2, the Gaussian envelopes of the Gabor patches were stationary while the carriers moved inside stationary envelopes. This type of motion is termed *local motion*. These local motions simulate that of a rigid-body contour-shape.

In different experiments, we varied (i) the motion direction and (ii) the temporal frequency of both the central contour and the surround texture adaptor. There were three adaptor conditions: center contour only, i.e. no surround (Fig. 2a), center and surround moving in the same direction (Fig. 2b), and center and surround moving in opposite directions (Fig. 2c). The test stimuli in all global motion conditions were pairs of single contours drifting in the same direction and with the same temporal frequency as the center contour adaptor (Fig. 2d). Example dynamic stimuli are illustrated in the Supplementary Information (Appendix B: Movies S1,S2,S3). We varied the temporal frequency of both center and surround contours. There were four values of temporal frequency: 0.9, 1.8, 2.7 and 3.6 shape cycles/sec. This resulted in 16 combinations of center/surround temporal frequencies (4 center × 4 surround temporal frequencies) that were measured for each of the three center/surround motion direction conditions (i.e. 4 no surround, 16 same and 16 opposite center-surround directions conditions), resulting in 36 conditions in total.

### Procedure

Each session started with an initial adaptation period of 90 s, followed by a repeated test of 0.5 s duration interspersed with top-up adaptation periods of 2.5 s to reinforce the initial adaptation. A schematic representation of the adapting and test procedure is shown in Fig. 2e. During the adaptation period, the shape-phase of the contour was randomly changed every 0.5 s in order to prevent the formation of afterimages and to minimize the effects of local orientation adaptation. The shape-phase of the test contour was also randomly assigned in every test period. The presentation of the test contour was signaled by a tone. Subjects were required to fixate on the marker placed between each pair of contours for the entire session. A head and chin rest helped to minimize head movements.

A staircase method was used to estimate the PSE. The geometric mean shape-frequency of the two test contours was held constant at 0.35 c/deg while the computer varied the relative shape-frequencies of the two tests in accordance with the subject’s response. At the start of the test period the ratio of the two test shape-frequencies was set to a random number between 0.7 and 1.44. On each trial subjects indicated via a button press whether the upper or lower test contour had the higher perceived shape-frequency. The computer then changed the ratio of test shape-frequencies by a factor of 1.06 for the first five trials and 1.015 thereafter, in a direction opposite to that of the response, i.e. towards the point of subjective equality or PSE. The session was terminated after 25 trials. We used a staircase method that was terminated after a fixed number of 25 trials, rather than a fixed number of reversals. This was done in order to keep the total amount of adaptation for each condition the same. The shape-frequency ratio at the PSE was calculated as the geometric mean shape-frequency ratio of the test that followed the lower shape-frequency adaptor to the test that followed the higher shape-frequency adaptor, averaged across the last 20 trials. Because the aftereffect is defined as the ratio of shape-frequencies at the PSE, its units are dimensionless. For each *with-adaptation* condition we made six measurements, three in which the upper adaptor had the higher shape-frequency and three in which the lower adaptor had the higher shape-frequency. In addition, we measured for each condition the shape-frequency ratio at the PSE in the absence of the adapting stimulus - the *no-adaptation condition*. To obtain an estimate of the size of the shape aftereffect we first calculated the difference between the logarithm of each with-adaptor shape-frequency ratio at the PSE and the mean of the logarithms of the no-adaptor shape-frequency ratios at the PSE. We then calculated the mean and standard error of these differences across the six measurements. These standard errors are the ones shown in the graphs.

## Additional Information

**How to cite this article**: Gheorghiu, E. and Kingdom, F. A. A. Dynamics of contextual modulation of perceived shape in human vision. *Sci. Rep.* **7**, 43274; doi: 10.1038/srep43274 (2017).

**Publisher's note:** Springer Nature remains neutral with regard to jurisdictional claims in published maps and institutional affiliations.

## Supplementary Material

Supplementary Information

Supplementary Movie S1

Supplementary Movie S2

Supplementary Movie S3

## Figures and Tables

**Figure 1:**
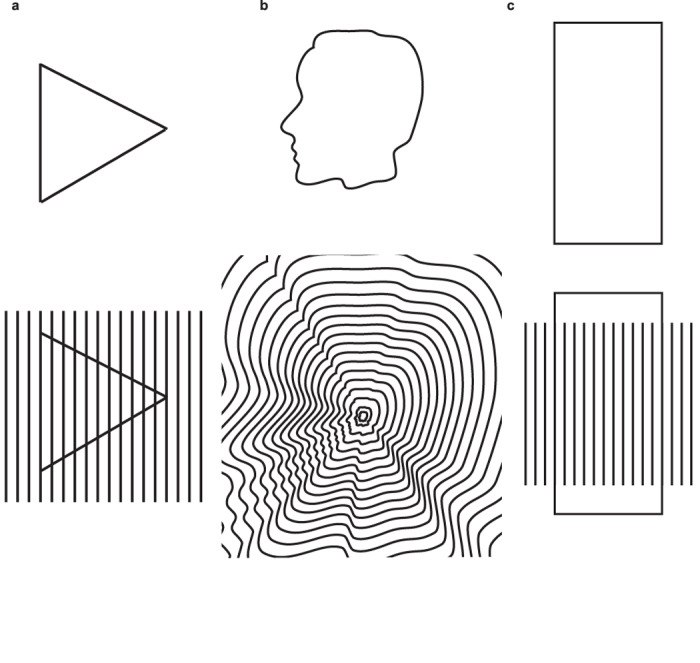
Examples of how texture impacts the perception of contour shape. (**a**) The three sides of the triangle are equally salient when presented in isolation (upper panel) but when surrounded by gratings (lower panel) the side parallel to the grating bars is perceived as a part of the grating not the triangle – example from Galli and Zama^11^. (**b**) A closed contour-shape (face profile, upper panel) flanked by several similar-shaped contours (lower panel) is difficult to be processed as an individual contour. (**c**) a larger rectangular shape partly embedded in surround gratings appears as being occluded by these gratings – example from Kanizsa^12^. Figure 1a and c from Gheorghiu, Kingdom and Petkov^8^ (used with permission of Elsevier).
