# Supplementary material for: Dynamics of contextual modulation of perceived shape in human vision
Source: Sci Rep. 2017 Feb 23;7:43274. doi: 10.1038/srep43274 (PMC5322363; doi:10.1038/srep43274)

Supplementary Information

Dynamics of contextual modulation of perceived shape in human vision

Elena Gheorghiu[1] & Frederick A.A. Kingdom[2]

[1] *University of Stirling, Department of Psychology, Stirling, FK9 4LA, Scotland, United Kingdom*
[2] *McGill Vision Research, Department of Ophthalmology, McGill University, Montreal, Qc, Canada*

Corresponding author: elena.gheorghiu@stir.ac.uk

Appendix A. Example Gabor element

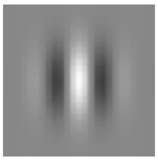

Our stimuli were contours and textures composed of strings of Gabor elements which are small patches of sinusoidal gratings (i.e. carrier) windowed by a smooth Gaussian envelope. The Gabor elements match a popular model of simple cell receptive fields (RFs) which are typically modelled with two-dimensional Gabor functions (see below). Gabor functions are a neurophysiological and mathematical sound model of simple cells RFs. A Gabor function has a number of parametric, well-studied properties such as spatial frequency, orientation, phase, contrast.

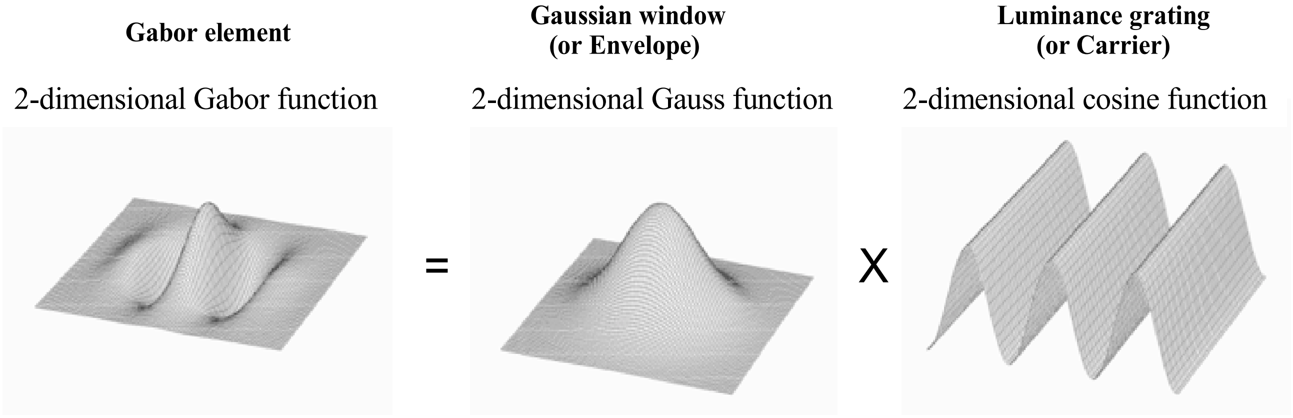

Appendix B. Example dynamic stimuli used in the motion direction experiment.

There were three adaptor conditions: center contour only, i.e. no surround (Movie S1), center and surround moving in the same direction (Movie S2), and center and surround moving in opposite directions (Movie S3). The test stimuli in all conditions were pairs of single contours drifting in the same motion direction and with the same temporal frequency as the center contour adaptor.

Movie S1: center contour only

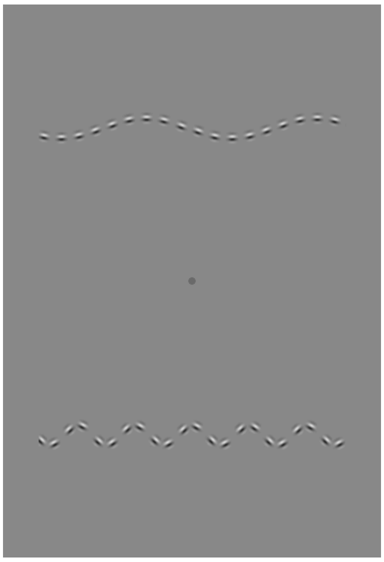

Movie S2: center and surround moving in the same direction

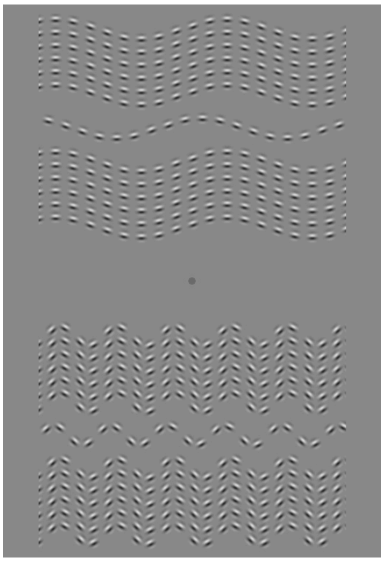

Movie S3: center and surround moving in opposite directions

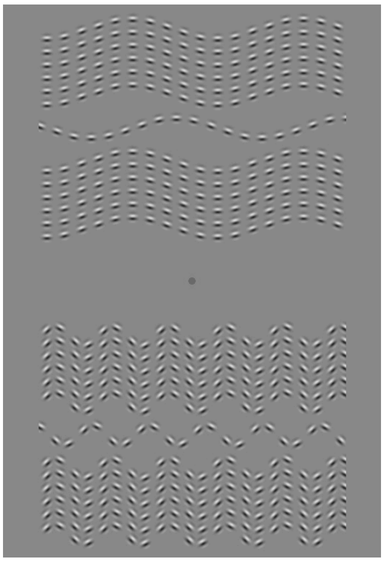

Supplement: Supplementary Information [file srep43274-s1.pdf]
